# Developing a Text Messaging Intervention to Reduce Deliberate Self-Harm in Chinese Adolescents: Qualitative Study

**DOI:** 10.2196/16963

**Published:** 2020-06-11

**Authors:** Suqian Duan, Haoran Wang, Amanda Wilson, Jiexi Qiu, Guanmei Chen, Yuqiong He, Yuanyuan Wang, Jianjun Ou, Runsen Chen

**Affiliations:** 1 Department of Psychiatry The Second Xiangya Hospital Central South University Changsha China; 2 Department of Psychological Medicine Institute of Psychiatry, Psychology and Neuroscience King's College London London United Kingdom; 3 Division of Psychology Faculty of Health and Life Sciences De Montfort University Leicester United Kingdom; 4 Department of Psychiatry University of Oxford Oxford United Kingdom

**Keywords:** text messaging, deliberate self-harm, adolescents, qualitative research, mental health

## Abstract

**Background:**

Deliberate self-harm is common during adolescence and can have detrimental consequences for the well-being of adolescents. Although it is sometimes difficult to engage adolescents in traditional psychotherapies for deliberate self-harm, SMS text messaging has been shown to be promising for cost-effective and low-intensity interventions.

**Objective:**

This study aimed to investigate the views of Chinese adolescents with deliberate self-harm about SMS text messaging interventions in order to develop an acceptable and culturally competent intervention for adolescents with deliberate self-harm.

**Methods:**

Semistructured interviews were conducted with 23 adolescents who had experience with deliberate self-harm. The transcripts of the interviews were analyzed using thematic analysis.

**Results:**

Four themes were identified: beneficial perception of receiving messages, short frequency and duration of messages, caring content in messages, and specific times for sending messages. Most of the participants perceived SMS text messaging interventions to be beneficial. The key factors that emerged for the content of the intervention included encouragement and company, feeling like a virtual friend, providing coping strategies, and individualized messages. In addition, the preferred frequency and duration of the SMS text messaging intervention were identified.

**Conclusions:**

Our study will help in the development of a culturally appropriate SMS text messaging intervention for adolescents with deliberate self-harm. It has the potential to decrease deliberate self-harm instances by providing acceptable support for adolescents with deliberate self-harm who may be reluctant to seek face-to-face psychotherapies.

## Introduction

Deliberate self-harm is defined as any nonfatal act of self-poisoning or self-injuries irrespective of the extent of suicidal intent or any other type of motivation [1]. The prevalence rate of deliberate self-harm is very high throughout adolescence, with percentages fluctuating between 12% and 23% [2]. Deliberate self-harm has been associated with a wide range of physical and psychiatric conditions, such as depression and suicidal behaviors, resulting in detrimental outcomes for the well-being of adolescents with deliberate self-harm [3]. Despite this, only a few interventions have been designed specifically for adolescents with deliberate self-harm, and of them, few have demonstrated promising therapeutic outcomes [2]. Thus, there is an urgent need to develop novel and acceptable interventions that target deliberate self-harm among adolescents.

One of the challenges in developing interventions for adolescents with deliberate self-harm is the reluctance of adolescents to engage with health professionals, especially in countries where mental health services are underdeveloped. Compared with traditional psychotherapies such as cognitive behavioral therapy, cost-effective and low-intensity treatment might be more feasible and acceptable among adolescents [4]. Recently, researchers have developed lower cost, higher coverage interventions, such as SMS text messaging interventions [5]. Texting is highly valued among adolescents nowadays, not only as a means of socializing but also as a way of providing and gaining peer support when facing difficulties in life [6]. Thus, SMS text messaging interventions might overcome some barriers of traditional psychotherapies and engage adolescents who are unwilling to attend face-to-face treatment [4].

Prior work has demonstrated the potential of SMS text messaging interventions as a cost-effective psychotherapy in the field of mood disorders, self-harm, and suicide-related behaviors with somewhat inconsistent findings [5,7]. For example, a randomized clinical trial suggested that participants who received SMS text messaging in the form of caring contact for 12 months had lower odds of experiencing any suicidal ideation than those who received standard care alone [5]. Furthermore, Hull et al [8] found that text interventions were effective in reducing symptoms of depression and anxiety to the extent of a diagnostic change. On the other hand, SMS text messaging interventions have had a significant effect on the likelihood or severity of current suicidal ideation [5] but has been found ineffective in improving depressive symptoms in another study [7]. Although the reasons behind these controversies remain unclear, all the studies have used very different content, and the frequency and duration of the text messages differed, which could partially explain the inconsistent findings. More importantly, none of these studies have developed interventions based on the perceptions and preferences of service users. According to Ram et al [9], service user engagement increases the possibility of developing interventions that are considered safe, have better usability, show they are clinically effective, and are culturally competent.

An important way of involving service users in the design of an intervention is to adopt a qualitative method, such as semistructured interviews or focus group discussions. Previous studies have used qualitative methods to involve service users in the design of SMS text messaging interventions [4,6]. However, these studies have been conducted in Western countries, and most of the participants were adults or mixed, meaning they did not look solely at adolescents. Given the cultural (West vs East) and population (adolescents vs adults) differences, developing tailored SMS text messaging interventions that are culturally competent and age specific is essential for generalizing this cost-effective intervention to different countries and adolescents.

Given the importance of involving service users in the design of an intervention, this study aimed to investigate the lived experiences of Chinese adolescents with deliberate self-harm regarding SMS text messaging intervention in order to develop an acceptable and culturally tailored intervention. Semistructured interviews were conducted to gather information from potential service users of an SMS text messaging intervention.

## Methods

### Participants

This study recruited participants from both the outpatient and inpatient units of the Second Xiangya Hospital, with special consideration given that inpatients were stable enough to participate. The inclusion criteria were as follows: (1) they must be between 12 and 17 years old, (2) they must have normal intelligence, (3) they should be able to complete the interview, (4) they have self-injured twice or more in the past 6 months, (5) they have no severe neurological or organic diseases, and (6) their parent or guardian understands the information sheet and agrees to fill in a consent form.

### Procedure

Participants were invited to participate in one semistructured in-depth interview with the researcher alone. At the beginning of the interviews, participants’ diagnoses of psychiatric disorders were confirmed with the clinical staff. Their demographic information and methods of self-harm were both self-reported and confirmed with the clinical staff. The interview started with an introduction of SMS text messaging interventions and some examples of text messages. These examples were designed based on SMS text messaging used in previous studies [6,10]. In addition, the examples were also discussed and reviewed by senior experts (clinical psychologists and psychiatrists) to tailor the texts for Chinese adolescents with deliberate self-harm. Example messages were only chosen if they were judged to be able to reduce the impulses of deliberate self-harm or negative emotions in a Chinese context. All example messages are listed in Textbox 1.

The interview was guided by open-ended questions and designed to understand the perceptions of participants on SMS text messaging interventions. The aim was to gain their perspectives on how to reduce their deliberate self-harm. An example of an interview question is *While you are having treatment for your self-harming behavior, we want to send you some text messages to help reduce your urges to self-harm. These messages could include information regarding how to deal with your low mood. How would receiving these messages make you feel?* The interview was developed based on questions identified during the literature review as well as previous text-base on interventions that have been shown to be effective [6]. All interviews were conducted in the psychotherapy room in the hospital by 3 authors (SD, JQ, and HW) with a clinical psychology background, who were trained by a senior clinical psychologist (RC). All interview questions and answers were communicated in Mandarin Chinese and then translated into English by the first author. Each interview lasted approximately 30 min; this was done to ensure that the participants did not become distressed. At the end of the interview, participants were asked to choose the messages they liked the most and the least from the sample provided.

**Examples of messages shown to participants at the interview.**

1. Nice to meet you! In the coming months, I will be honored to share your joy and pain. From today, please treat yourself as if you were someone else, and do some sweet little things for yourself, such as: light candles in the room, and find the smell that makes you happy. If you want to get more help for self-injury contact us. Have a good day!
2. What lies behind you and what lies before you are tiny matters compared to what lies within you. Have faith in yourself and success can be yours. Hope you have a lovely week.
3. Hello. When you are in a bad mood, you may think: “I have experienced many difficult situations and I survived, this time is no exception.” Hope you have a good day!
4. Letting go of resentment is a gift you give yourself, and it will ease your journey immeasurably. Make peace with everyone and happiness will be yours. Hope everything is going well with you.
5. If you want to self-harm, you may try the following things instead: (1) Draw the person or thing you hate on the balloon and then pop it. (2) Write a letter to someone you hate or who hurts you, talk about what they did to you and why you hate them. Save the letter and read it later. I hope your life is getting better and better
6. Pay attention to activities that have a positive impact on your mood. Note these activities and refer to them when you hit a low point to improve your mood. Remember to take good care of yourself!
7. Hi! Remember, doing something pleasant is the best way to get rid of pain. You may try the following things: (1) Eat chocolate or your favorite food. (2) Do something exciting, such as learning a sport. (3) Take a movie or video with your camera. Hope you have a good day!
8. Hi, do you remember me? If you are in a low mood, you may be able to call the self-injury crisis hotline and talk to people. The number is: 4001619995. I hope everything is OK recently! Don’t worry about yesterday and tomorrow, we only live for today! Wish you a happy life!
9. Today is your birthday. Happy birthday to you! In order to reward yourself for overcoming difficulties and treating yourself well in the past year, you may go to your favorite coffee shop for tea or coffee with your friends.

### Data Analysis

All interviews were audio recorded and then transcribed verbatim by 2 authors (SD and JQ). The data were then analyzed using thematic analysis. According to Braun and Clarke [11], there are 5 phases included in this approach. First, 2 authors familiarize themselves with the transcripts by independently reading and rereading them. Second, the initial codes of the transcripts are generated. Third, themes are identified from the transcripts. Fourth, the 2 authors share their themes and compare them with each other. Fifth, a final consensus on the themes is achieved between the 2 authors to decide how to define and name themes [12,13]. After this, the 2 authors engaged in a discussion with a senior investigator (RC), and the senior investigator read over all the themes and subthemes to assess the reliability of the findings.

### Ethics Approval

This study protocol was approved by the Ethics Committee of the Second Xiangya Hospital of Central South University.

### Availability of Data and Materials

The datasets used and/or analyzed during this study are available from the corresponding author upon reasonable request.

## Results

### Participants

A total of 23 patients at the Psychiatry Department of the Second Xiangya Hospital were interviewed. The average age of the participants was 15 years, ranging from 12 to 17 years. The participants were predominantly female. Demographic information on participants and their methods of deliberate self-harm are summarized in Table 1. Out of the 23 participants, 21 were diagnosed with depression. Of these, 1 had both depression and anxiety, and 1 had depression with psychotic symptoms. Moreover, 2 participants had bipolar disorder. Regarding the methods of self-harm, almost all participants had reported cutting themselves. Other methods of self-harm include pinching, head banging, biting, scratching, punching the wall, asphyxiation, burning, dousing head with cold water, poisoning, strangling, and hair pulling. Of the participants, 13 used an average of 2 to 3 methods for self-harming.

**Table 1:** Participant characteristics.

Participant	Age (years)	Gender	Diagnosis	Methods of self-harm
1	16	Female	Depression	Cutting
2	14	Male	Bipolar disorder	Cutting
3	14	Female	Depression	Cutting and scratching
4	16	Female	Depression	Cutting and head banging
5	16	Female	Depression and anxiety	Punching the wall
6	15	Female	Depression	Cutting
7	13	Female	Depression	Cutting and asphyxiation
8	16	Female	Depression	Cutting and burning
9	16	Female	Depression	Cutting
10	15	Female	Bipolar disorder	Cutting and head banging
11	17	Female	Depression	Cutting and dousing head with cold water
12	14	Female	Depression with psychotic symptoms	Cutting and head banging
13	16	Female	Depression	Cutting
14	12	Female	Depression	Cutting
15	17	Male	Depression	Cutting
16	15	Male	Depression	Cutting and poisoning
17	16	Female	Depression	Cutting, biting, and pinching
18	15	Female	Depression	Cutting and pinching
19	17	Female	Depression	Cutting and head banging
20	17	Female	Depression	Cutting, biting, and strangling
21	15	Female	Depression	Cutting, pinching, and hair pulling
22	14	Female	Depression	Cutting and pinching
23	15	Male	Depression	Cutting and pinching

### Choice of Sample Messages

Several participants chose messages 7 and 9 as the messages they liked the most. Messages 5 and 8 were also chosen by some participants, whereas only a few participants chose messages 3, 4, and 6. In addition, 4 participants did not choose any message as their favorite. Message 4 was the least liked message among the majority of participants. Several participants did not like messages 2 and 3 as well. The number of participants and their choice of each message are summarized in Table 2.

**Table 2:** Number of participants and their choice of messages.

Message no.	Content of the message	Participants who liked the message, n	Participants who disliked the message, n
1	Treat yourself well	0	5
2	Have faith in yourself	0	8
3	You will survive	2	6
4	Let go of resentment	1	10
5	Try different things to DSH^a^	5	4
6	Pay attention to positive things	1	3
7	Suggestions for activities	9	3
8	Self-harm hotline	5	4
9	Birthday greeting	9	4
N/A^b^	Did not choose any message	4	0

^a^DSH: deliberate self-harm.

^b^N/A: not applicable.

### Thematic Analysis

The following sections represent the perceptions of the adolescents on SMS text messaging interventions in further detail. Four themes were identified from the transcripts of the semistructured interviews: (1) beneficial perception of receiving messages, (2) short frequency and duration of the messages, (3) caring content in messages, and (4) specific times for sending messages. Each section starts with the name of the theme. The extracts that best represent the theme are then presented and discussed.

#### Theme 1: Beneficial Perception of Receiving Messages

Most participants mentioned that the SMS text messaging intervention could be beneficial for them and were willing to receive text messages. Several of them said that the messages could help them have prompt self-reflection and stay calm:

...I hope to receive this type of messages because it helps me think about myself and my recent behaviours. It may ask me to take good care of myself, so I think I should treat myself well...

p13

Other participants said that it could be helpful to know what resources are available, such as research-based information or a contact number of someone whom they could talk to when they want to self-harm, which might reduce their urges to self-harm:

...Having some scientific understanding of my symptoms via text-messages could be effective for reducing my self-harm. It is also helpful to have some useful information such as contact number of people who are there to help...

p16

However, some participants also said that they would not like a contact number because they would not know the reliability of this information. In addition, there were also a few participants who doubted the benefit of the intervention, as they believed the messages would not improve their depressed mood to a large extent:

...I am not sure whether text-messaging intervention will be helpful or not. Reading these messages might be boring for me, but I believe it does no harm...

p22

...When I read the messages, I will not have any particular feeling towards it, as I have read too much information similar to this. In addition, if there is information regarding certain website or certain number, I will think they are trying to advertise or blackmail me...

p19

Furthermore, a small number of participants believed that they would not find this intervention useful because their parents keep their mobile phones for them most of the day. However, they mentioned that the intervention might also be helpful if their parents receive these messages.

#### Theme 2: Short Frequency and Duration of Messages

Participants had different suggestions for the frequency of text messages. Most participants agreed that it would be great to receive the messages more often than once a week but less often than once a day. This frequency would allow them to pay sufficient attention to the content of the messages:

...I prefer to receive this type of messages every three days. If I receive them too often, I will not want to read them...

p9

...I am not sure about the frequency, but I would say do not be too frequent. Receiving messages once every two to three days might be good...

p22

A few participants mentioned that they would like to receive messages more often than once a day, with 1 participant specifically mentioning 1 message each during the day and night. Importantly, 2 participants mentioned that text messages could be more frequent at the beginning, and then the frequency should reduce with time:

...Just keep sending the messages at the beginning of this intervention (with a high frequency). The frequency can decrease after some time....

p1

In addition, some participants answered that they did not care about the frequency of the messages very much because the messages would be equally effective regardless of how often they receive the messages.

Regarding the duration of the intervention, many participants said it would be great to receive these messages for a duration of 2 months to 6 months. As they suggested, if the duration is too long, they would no longer pay attention to the contents. Moreover, the effectiveness might be reduced if the duration is too short:

...I think three-month time will be great for me, if I receive these for a longer duration, I will not read them...

p9

...I suggest to send messages for one to two months, but it also depends on how long the patient recover from the symptoms...

p19

Nevertheless, 2 participants suggested a long duration (1 year) and a short duration (1 month). Furthermore, 2 participants said that the duration could be as long as possible, as they would not want to stop receiving the messages:

...I would like to receive these messages for a duration as long as possible, if I stop receiving these messages one day, I will feel sad and empty...

p1

#### Theme 3: Caring Content in Messages

There are 4 key factors identified in the caring content of messages: encouragement and company, feeling like a virtual friend, providing coping strategies, and individualized messages.

Many participants mentioned that they would like to receive messages as a form of social support. This is important, as it can make them feel encouraged and less isolated, which could potentially improve their depressive symptoms and low mood:

...The messages I want to receive may include any words for encouragement, help and comfort. These will make me feel supported and have the strength to fight with my disease...

p2

...I need some encouragement and sense of being cared for in my life, so if the messages could include this type of information, I might feel better...

p5

I cannot think about any particular type of messages, but I would say ‘I will always be with you’ will be helpful for me, it makes me feel less lonely...

p7

...I guess something like “you are not alone” in the messages will make me feel better...

p11

Although the participants used a tentative language, it was clear, regardless of whether they received a text message or not, that feeling lonely was at the core of deliberate self-harm. They wanted stable, long-term caring relationships to resolve this.

Some participants said that the messages should make them feel like they were sent by their friends, rather than by psychologists, parents, teachers, or an authority. Imagining that they were receiving messages from friends would make them feel less lonely. On the other hand, if the messages are too *official*, they might feel bored and might not want to look at the messages:

...Don’t include messages that make me feel like my parents are teaching me something, I need encouragement from a friend, not from a teacher or parent...

p10

...The messages should not be very official. Instead, they need to be in the simple languages we use in daily lives. It is even better if it can make me feel like talking with a friend....

p23

A few participants suggested that the messages should include coping strategies and knowledge of self-harm. This is important for them to understand why they start self-harming and what they can do to prevent their self-harm:

...It is good for the messages to include some coping strategies for self-harm in my daily life. It will be very helpful if I receive this type of thing before I want to harm myself...

p19

...The messages should have a list of coping strategies in simple languages. I will read them if I am interested...

p22

...If they (the messages) let me know how I could deal with my stress; this might be useful...

p2

Participants mentioned that the intervention could be more effective if the messages were tailored to give them a further sense of coming from someone caring. Participants specifically mentioned that generic messages that are sent to everybody would not work for them:

...I will enjoy reading these messages if I know they are tailored for me, rather than generic messages sent to everybody who has similar conditions...

p23

...I will be very interested in the content of the messages if they are sent ‘one to one’, otherwise, I guess it does not have any personal meaning for me...

p8

#### Theme 4: Specific Times for Sending Messages

As mentioned by the participants, receiving the messages can reduce their urges to self-harm. Most participants stated that their urges to self-harm were the strongest in the evening, especially when they were alone. However, a few participants also said that they harmed themselves in the afternoon or morning. A small number of participants stated that there was no fixed time for conducting self-harm; they would harm themselves whenever they felt depressed or irritable:

...I normally want to harm myself in the evening when I am alone, I just want to hurt myself because I feel sad...

p2

...There was no fixed time for me to hurt myself. I will do it whenever my mood bursts...

p3

Regarding how self-harm related to their life events, most participants said that they would self-harm when stressful life events occurred. These included being bullied, divorce of parents, difficult peer relationships, break-up in a romantic relationship, feeling unsupported, and feeling stressed from schoolwork. In addition, several participants identified that their urges to self-harm would increase before they return to school after the summer holidays. Nevertheless, 3 participants could not think of any particular events that triggered their self-harm:

I hurt myself when bad things happened in my life, such as something that makes me feel I am not loved by my parents and when I fail to have good performance in exams...

p1

...I start to self-harm mainly after some bad things happened, such as being told off by my parents, being bullied in school etc...

p2

...I will cut myself when I feel stressed at school and after I argue with my parents...

p23

I harm myself every time I break up with my girlfriends, the severity of my self-harm depends on how much I care about her...

p4

## Discussion

### Principal Findings

To our knowledge, this is the first study to investigate the views of adolescents with deliberate self-harm on SMS text messaging interventions to develop an acceptable and culturally tailored intervention. There were 23 patients interviewed at the psychiatric department who met the inclusion criteria. The analysis showed that by using a qualitative approach, most of the participants perceived the intervention as beneficial for them. In addition, the preferred frequency and duration of an SMS text messaging intervention were identified. It was found that the key time for delivering the messages is during the evening and after stressful events or during a crisis. Four key factors emerged for the content of the intervention: encouragement and company, feeling like a virtual friend, providing coping strategies, and individualized messages. On the basis of these results, SMS text messaging interventions tailored for Chinese adolescents will be developed, and randomized controlled trials assessing the feasibility and effectiveness of this intervention will be planned by our research team.

### Perception of Receiving Messages

Despite different cultural contexts, the perception of SMS text messaging interventions is somewhat similar within the Western and Chinese cultures. Consistent with Owen et al [6], we found a broad range of support among service users for using text messages as an intervention for deliberate self-harm. Nevertheless, compared with Western studies, some participants in our study were hesitant toward receiving the SMS text messaging intervention, as they considered the effectiveness of this intervention unknown or questionable. Although the clear reason behind this is unknown, it could be related to the generally low engagement with and low level of understanding of mental health services among Chinese adolescents.

### Frequency and Duration of Messages

Few previous studies have set out to understand what the appropriate frequency and duration are for SMS text messaging interventions from a service user’s perspective. Nevertheless, in a study by Larsen et al [4], participants indicated that frequent, but not too frequent, messages would be useful. This is consistent with our findings that most participants suggested a frequency of more than once a week but less often than once a day. In addition, our participants proposed a duration of intervention that is short rather than long, with most of them suggesting 2 to 6 months. This duration has been employed in most SMS text messaging interventions in the literature [14], and our findings provide supporting evidence for this design, confirming its acceptability from a service user’s perspective.

### Content in Messages

Considering the content of a text messaging intervention is the most important step when designing the intervention. Compared with research conducted within the Western culture, our findings have demonstrated both consistency and novelty. To illustrate, our analysis identified some similar factors as those identified by 2 studies [4,15], such as encouragement and coping strategies to deal with deliberate self-harm. In addition, the factor *individualized messages* has also been identified by Owen et al [6], in which service users rejected the idea of a generic, one-size-fits-all approach for text messages. The consistency of these findings may indicate the importance of these factors in designing SMS text messaging interventions regardless of the cultural contexts of the service users. On the other hand, some factors have not been found in previous studies, such as feeling like a virtual friend. Thus, this finding could be specific to the Chinese cultural background and the population of our service users (adolescents). Adolescence is the period of time when peer relationships and friendships are increasingly established and valued [16]. When facing difficult situations, adolescents are more likely to seek help from their friends rather than from their teachers or parents. This phenomenon might be more common in China, as Chinese parenting has often been described as *authoritarian* or *controlling* [17]. In addition, loneliness of adolescents has been associated with a range of mood disorders co-occurring with deliberate self-harm, such as depression [16]. As such, seeking social support from a friend can be especially important and helpful for Chinese adolescents with deliberate self-harm. Having related content in text messaging interventions could also be beneficial for Chinese service users.

### Time for Sending Messages

Previous studies of text messaging interventions have not considered the time of the day when the text messages should be delivered [5,7,14]. Our study, therefore, tried to identify particular times of day when the urges of participants to self-harm were the strongest, and situations in which the likelihood of engaging in deliberate self-harm were high. Although some of our participants did not have a fixed time for self-harming, most of them conducted deliberate self-harm during the evening, especially when they were alone. This suggests that more messages could be sent during the evening to reduce the impulses of deliberate self-harm at the right time. In addition, we found that different types of stressful life events such as the divorce of parents and break-up in a romantic relationship could trigger deliberate self-harm among Chinese adolescents. In order to tailor the intervention for individuals, it might be beneficial to send the messages after certain stressful life events have occurred or when they are in crisis.

### Limitations

There are a few limitations in the study that warrant discussion. First, the participants in our study were predominantly adolescent girls. As a result, our findings may be more representative of how girls with deliberate self-harm think about text messaging interventions. Although deliberate self-harm shows a higher female-to-male ratio [18], future studies involving a more balanced gender ratio are needed. Second, although 1 participant mentioned that an SMS text messaging intervention does no harm, the interviewer did not specifically ask questions regarding the potential negative effects of such interventions. Nevertheless, this could be an important theme to be mindful of in future research to avoid any adverse outcomes of SMS text messaging interventions. Third, our findings might be generalizable to Chinese adolescents with deliberate self-harm only to a certain extent, as all of our participants had been diagnosed with psychiatric disorders (eg, depression). It would be meaningful to conduct similar research with participants who do not have a clinical diagnosis or who have less severe conditions. Finally, it is worth bearing in mind that China is a big country with 56 ethnic groups, a diverse culture, and a mix of rural and urban areas. This implies that adolescents who were born in different areas in China and belong to different ethnic groups might have different attitudes toward SMS text messaging interventions, which have not been considered in our study.

### Conclusions

Our study is the first to investigate the views of adolescents with deliberate self-harm on text messaging interventions. It can be used to inform the development of a culturally tailored SMS text messaging intervention for adolescents with deliberate self-harm. The study has the potential to decrease instances of deliberate self-harm and provide cost-effective, low-intensity, and acceptable support for adolescents with deliberate self-harm who may be reluctant to seek face-to-face psychotherapies.
